# Tubastrine, an antioxidant molecule from *Tubastraea tagusensis* sun coral, in the reversion of oxidative stress and neuron's death induced by Aβ42

**DOI:** 10.1111/jcmm.70165

**Published:** 2024-12-20

**Authors:** Amanda G. Silva, João Vitor Cerávolo Rostirola, Filipe Duo Speri, Juliana Guanaes Pina, Marcelo V. Kitahara, Giovanna B. Longato, Juliana M. Sciani

**Affiliations:** ^1^ Laboratório de Produtos Naturais Universidade São Francisco Bragança Paulista Brazil; ^2^ Unidade Integrada de Farmacologia e Gastroenterologia (UNIFAG) Bragança Paulista Brazil; ^3^ Centro de Biologia Marinha Universidade de São Paulo São Sebastião Brazil

**Keywords:** Alzheimer's disease, antioxidant, coral molecules, oxidative stress, treatment, tubastrine

## Abstract

Alzheimer's disease (AD) is a progressive neurodegenerative disorder involving mitochondrial dysfunction and consequent production of reactive oxygen species (ROS), generated after amyloid peptide (Aβ42) accumulation. In this study, we isolated a new antioxidant molecule from the sun coral *Tubastraea tagusensis* and analysed it in cells exposed to oligomeric amyloid‐beta peptide 1‐42 (oAβ42). The coral was collected and immersed in methanol for the release of compounds, which were submitted to antioxidant DPPH and FRAP activity‐guided fractionation using solid‐phase extraction and HPLC. An active pure molecule was analysed by mass spectrometry and tested in SH‐SY5Y differentiated neurons previously exposed to 5 μM oAβ42. The isolated active molecule was identified as tubastrine, which could significantly inhibit the cell death caused by the amyloid peptide. Moreover, oAβ42 increased the percentage of ROS in neurons‐like from 40% to 65%, and the treatment with tubastrine reduced it to 50%. The antioxidant power of neurons‐like after oAβ42 decreased significantly, while the compound reversed it, reaching similar values to the untreated cells. Therefore, tubastrine can reverse an important pathophysiological mechanism of AD, oxidative stress, by increasing neuronal antioxidant power and reducing ROS levels, able to prevent neuron‐like cell death caused by oAβ42.

## 1 INTRODUCTION

Oxidative stress is an imbalance between the generation of reactive oxygen species (ROS) and antioxidant defences.^1^ ROS and reactive nitrogen species (RNS) are known as highly unstable free radicals that consist of an atom with unpaired electrons in its orbit. Examples are hydroxyl radical (OH^•^), superoxide anion (O_2_^•−^), hydrogen peroxide (H_2_O_2_) and singlet oxygen (^1^O_2_). Their overproduction, predominantly by mitochondria, results in a deleterious process that cause damage in lipids, proteins, DNA, and membranes, ultimately inducing cell death.^2^

This condition is related to several diseases, including Alzheimer's disease (AD), which is a progressive neurodegenerative disorder, common aging disease and one of the leading causes of dementia, responsible for up to 80% of all its diagnoses.^3^, ^4^ In 2018, the estimated global prevalence of dementia was 50 million people (Alzheimer's Disease International, 2018), and it progressively increases with age.^3^ As a result, AD is rapidly becoming one of the most costly, lethal, and burdensome diseases of the 21st century.^5^

The main cause of AD is the deposition of oligomeric amyloid‐ beta peptide 1‐42 (oAβ42), generated after enzymatic processing of the amyloid precursor protein. Aβ1‐40 is present in its normal soluble isoform, but a change in the cleavage pattern (after a mutation, for example) can lead to Aβ1‐42 production, which readily aggregates and forms amyloid plaques,^6^ potentially toxic to neurons.

Amyloid‐ beta peptide 1‐42 initiate several intracellular mechanisms, and one of them is a disorder of autophagy‐lysosome system. Lysosomes are responsible for the intracellular degradation of endocytosed components through autophagy, but in the context of AD, dysfunction of this process can lead to harmful accumulation of Aβ in the brain.^7^ Such accumulation is not solved by the ubiquitin‐proteasome complex, which is inhibited in the disease.^8^

Moreover, the increased permeability of lysosomes facilitates the leakage of compounds that bind to iron ions, which are released from the mitochondria, compromising cellular respiration. Mitochondria, in turn, release ROS and several factors that activate caspase and consequently apoptosis. The oxidative stress breaks DNA, contributing to the cell death, and the degeneration process of the brain.^9^

Nowadays, there is evidence that not only ROS, involved in oxidative damage, can modulate the production/secretion of Aβ, but it can also reciprocally promote excessive ROS generation, leading to a vicious cycle.^10^ Based on this, compelling evidence of the oxidative/antioxidant imbalance in AD has led to the formulation of a hypothesis that compounds absorbing free radicals and/or boosting defence mechanisms against oxidative stress could provide therapeutic benefits in this disease.^11^ Thus, various antioxidants, such as curcumin, resveratrol, vitamins E and C, and alpha‐lipoic acid, have been tested for their potential to preserve or improve cognitive performance in patients with mild cognitive impairment.^12^, ^13^ Moreover, the research on biomolecules from marine organisms have already uncovered the potential of several of them to be used in a variety of diseases.

Biodiversity offers a wide library of molecules, which can be used for the obtention of new structures or biological activities.^14^ Several commercially available drugs have been derived from compounds found in marine animals, demonstrating the potential of marine sources for the discovery of novel molecules. One example is ziconotide (commercially available as Prialt®, TerSera Therapeutics), a peptide drug used to treat chronic pain, which was originally isolated from the cone snail *Conus magus*.^15^ Another is Trabectidin (Yondelis®, Janssen‐Cilag and PharmaMar) from the sea squirt *Ecteinascidia turbinate* and Cytarabine (Ara‐C) from the Caribbean sponge *Cryptotheca crypta* for the treatment of advanced soft tissue sarcoma and leukaemia, respectively.^16^, ^17^ Although potentially holding the secrets for drug discovery, marine animals, especially those that bear's secretions and venoms, are still poorly explored.

We studied the Atlantic invasive scleractinian coral *Tubastraea tagusensis*, that invaded the Atlantic and, nowadays, are widespread along the Brazilian coast. From its extract, we used an activity‐guided fractionation to obtain a new antioxidant molecule—tubastrine—able to scavenge radicals from biochemical assays, as well as in SH‐SY5Y differentiated cell exposed to oAβ42, with reduction of cell death.

## 2 MATERIALS AND METHODS

### 2.1 Sample acquisition

*Tubastraea tagusensis* specimens were collected in São Sebastião (ICMBio licence number 68917‐1) and maintained in filtered seawater. The extraction was conducted as previously reported by our group and the literature, with slight modifications.^18^, ^19^ About two polyps of the colony were placed in methanol for 24 h, and the content secreted was collected, centrifuged, and the solvent dried with a rotary evaporator. The extract was then stored at −20°C.

### 2.2 Fractionation

The aliquots of the extracts were re‐suspended in water containing 0.1% trifluoroacetic acid and subjected to solid‐phase extraction (SPE) using C18 cartridges (3 mL, 475 m^2^/g, 500 mg, 20 μm porosity, Supelclean™ LC‐18 SPE Tube, Supelco®), with elution by 0, 20, 40, 60, 80, and 100% acetonitrile in water containing 0.1% trifluoroacetic acid.

The active antioxidant fraction (SPE 20%) was then subjected to high‐performance liquid chromatography (HPLC) (1260 Infinity, Agilent Technologies, Santa Clara, CA, USA) coupled to a C18 column (Ascentis 250 × 4.6 mm, 5 μm, Supelco). The elution was performed using a 0% to 100% linear gradient of solvent B over A (where B corresponds to a solution of 90% acetonitrile (ACN) containing 0.1% trifluoroacetic acid, and A is a solution of 0.1% trifluoroacetic acid), for 30 min, at a constant flow rate of 1 mL/min and monitored by a UV detector at wavelengths of *λ* = 214 and 254 nm. The fractions were manually collected and tested for antioxidant activity.

### 2.3 Molecule identification

The isolated and active molecule was analysed by mass spectrometry for identification. The sample was inserted into an ESI‐Xevo TQ‐S (Waters Co., USA), without column, with elution with ACN 50% containing 0.1% formic acid, at a constant flow rate of 0.2 mL/min. The spectrum was acquired under positive ionization mode, with source temperature at 115°C, desolvation temperature of 500°C, desolvation gas flow of 800 L/h, and capillary and cone voltage of 3 and 30 kV, respectively. The ion 194 m/z was selected for fragmentation with argon gas.

Equipment control and data acquisition were performed using MassLinx 4 and the fragmentation profile was manually compared to the literature.

### 2.4 Radical scavenging activity

#### 2.4.1 DPPH test

For the evaluation of the antioxidant activity of the extract, SPE, or HPLC fractions, the DPPH (2,2‐Diphenyl‐1‐picrylhydrazyl) assay was used. This test was conducted in 96‐well microplates, where 10 μL of sample (1 mg/mL for fractions and 260 μM for the isolated molecule) was incubated with 10 μL of DPPH (0.1 mM final concentration, diluted in methanol, Sigma‐Aldrich, St Louis, MO, USA) and added to 180 μL of methanol. The reaction was incubated for 5 min at room temperature. After this period, absorbance was measured using a spectrophotometer (GloMax® Discover Microplate Reader spectrophotometer, Promega, WI, USA) at a wavelength of 515 nm. The assays were performed in triplicate, and the mean of the obtained values was considered for calculation.

As a control, 10 μL of methanol was added instead of the sample, and as the assay control, ascorbic acid (1 mM, Sigma‐Aldrich, St Louis, MO, USA) was used. The absorbance value obtained with ascorbic acid (assay control) was not used for calculation.

For the calculation of antioxidant activity, as a percentage, the following formula was used:

100 − ((A_sample_ × 100)/A_control_).

#### 2.4.2 FRAP test

For the FRAP (ferric reducing antioxidant power) assay, phosphate‐buffered saline (PBS 50 mM, pH 7.3) was incubated with 1% potassium ferrocyanide and sample (1 mg/mL for fractions and 260 μM for the isolated molecule) for 20 min at 50°C. Afterward, 10% trichloroacetic acid was added, and the solution was centrifuged at 3000 rpm for 10 min. An aliquot was added to distilled water and 0.1% ferric chloride, and the solution's absorbance was measured in a GloMax® Discover Microplate Reader spectrophotometer (Promega, WI, USA) at *λ* = 700 nm. The relative percentage of reducing power was calculated as

$$\left(A_{\text{sample}}-A_{\text{control}}\right)/\left(A_{\text{standard}}-A_{\text{control}}\right)\times 100$$

A control (buffer in place of the sample) was added, and ascorbic acid was used as a reference standard. The samples were analysed in duplicate, and the results are presented as % antioxidant activity. All reagents were purchased from Sigma‐Aldrich (St Louis, MO, USA).

### 2.5 Neuron's model of AD

SH‐SY5Y cells (ECACC, Sigma‐Aldrich, St Louis, MO, USA) were grown in DMEM/F12 cell culture media (Gibco Life Technologies, Grand Island, NY, USA) containing 10% fetal bovine serum (LGC biotechnology, Cotia, SP, Brazil) and 1% antibiotic (penicillin/streptomycin, Gibco Life Technologies, Grand Island, NY, USA). After confluence, trypsin–EDTA 0.25% (Gibco Life Technologies, Grand Island, NY, USA) was added to remove cells, which were placed in a 96‐well plate (1 × 10^4^/well) and then the cells were differentiated to neuron‐like by changing the culture media to DMEM/F12 media containing 1% fetal bovine serum (LGC biotechnology, Cotia, SP, Brazil), 0.1% antibiotic (penicillin/streptomycin, Gibco Life Technologies, Grand Island, NY, USA), and 10 μM retinoic acid (Sigma Aldrich, Saint Louis, MO, USA). This media was replaced each 2 days, until 8 days. After the completion of differentiation, the cells were incubated with 5 μM oligomeric amyloid‐ beta peptide 1‐42 (oAβ42) (GenScript, Piscataway, NJ, USA), and after 48 h, cells were treated with 100 μM of tubastrine (diluted in sterile PBS) for 24 h (cells in the control group were treated only with sterile PBS). After this period, cells were submitted to antioxidant activity and cell viability analysis, as described below.

### 2.6 Total antioxidant activity in cells

#### 2.6.1 Antioxidant power by ABTS test

The tubastrine was evaluated in its ability to inhibit the oxidation of ABTS (2,2′‐azino‐di[3‐ethylbenzthiazoline sulphonate]) by metmyoglobin, compared to Trolox, a water‐soluble tocopherol analog.

After treatment with oAβ42 and tubastrine, neurons‐like were detached with trypsin–EDTA 0.25% (Gibco Life Technologies, Grand Island, NY, USA) and collected by centrifugation (1000×*g* for 5 min). After being placed in a PBS buffer, they were disrupted by sonication at low temperature. The supernatant of centrifugation (10,000×*g* for 10 min at 4°C) was collected and used for the assay, in triplicate, performed following the manufacturer's instructions (Antioxidant Assay Kit, 709,001, Cayman Chemical).

#### 2.6.2 ROS measurement by DCFH‐DA probe

After exposure to oAβ42 (5 μM) for 4 h and tubastrine (100 μM) for 24 h, each 48 of the wells containing neurons‐like was incubated with 1 μL of a DCFH‐DA solution (10 μM, Sigma‐Aldrich, St Louis, MO, USA). The plates were kept in a humidified incubator at 37°C, 5% CO_2_, protected from light. After 45 min, the supernatant was removed, and the cells were washed with Hank's buffer (Hanks balanced salt solution, NaHCO_3_: 0.35 g/L, and glucose: 1.0 g/L), and dissociated from the plate using trypsin–EDTA 0.25% (Gibco Life Technologies, Grand Island, NY, USA). The cells were then resuspended in DMEM/F12 medium and transferred to microtubes, which were centrifuged at 1500 rpm for 5 min. The supernatant was discarded, and the precipitated cells were resuspended with Hank's buffer and transferred to flow cytometry tubes. The fluorescence emission of the cells was acquired using a Guava® easyCyte 5HT flow cytometer (Millipore, MA, USA), with a green laser. Population analysis (5000 events) was performed using the software InCyte 2.2.2, in triplicate.

### 2.7 Cell viability

For MTT, which evaluates the mitochondrial activity, the supernatant was removed, and the MTT solution (Sigma‐Aldrich, 0.5 mg/mL), diluted in DMEM/F12 cell culture media (Gibco Life Technologies, Grand Island, NY, USA), was added and the plate incubated in a humidified incubator (37°C, 5% CO_2_) for 3 h. The MTT solution was later discarded, and 100 μL of dimethyl sulfoxide was added to each well to dissolve the formazan crystals. The absorbance was measured on a spectrophotometer (GloMax® Discover Microplate Reader, Promega, Madison, WI, USA) at a wavelength of 540 nm. Cell viability was defined as the percentage of absorbance observed in the treatments relative to the absorbance of the respective cells in the negative control group (100%), which consisted of incubation with PBS instead of the samples, in triplicate.

To verify the cell membrane integrity after treatment, SH‐SY5Y were washed three times with sterile PBS and incubated with 0.1 μM of calcein‐AM (Invitrogen/ Thermo Fisher Scientific, Waltham, MA, USA) for 45 min in a humidified incubator at 37°C and 5% CO_2_. After incubation with the probe, the supernatant was discarded, and the cells were washed with sterile PBS. The fluorescence emission of the cells was acquired using a Guava® easyCyte 5HT flow cytometer (Millipore, MA, USA), with a green laser. Population analysis (5000 events) was performed using the software InCyte 2.2.2, in triplicate.

### 2.8 Statistical analysis

The results were expressed as mean ± standard error of the mean. In the neurons‐like experiment, one‐way analysis of variance (ANOVA) statistical analysis with a post hoc Tukey was employed to compare all groups using GraphPad Prism v 8.3.0. Differences were considered significant when *p* < 0.05 between the control and oAβ42 groups (shown as *) or to between oAβ42 and oAβ42 + tub groups (shown as #).

### 2.9 Molecular docking

Molecular docking experiments were conducted using Swiss Dock webserver (http://www.swissdock.ch), with AutoDock Vina docking engines. The ligand was provided as SMILES (N=C(N)N/C=C/c1ccc(O)c(O)c1) and the target with Protein Data Bank (PDB) code, as follows: Superoxide Dismutase (8GSQ‐chain A), Catalase (1DGB‐chain A) and Glutathione peroxidase 3 (2R37‐chain A). All proteins were obtained by x‐ray and from human. The box center was positioned in the active site of each protein, and the box size was set in 20x–20y–20z Å. The sampling exhaustivity was set in 4 and considering hydrogen bonds and ionic interactions.

## 3 RESULTS

### 3.1 Radical scavenging activity‐based fractionation

**FIGURE 1:**
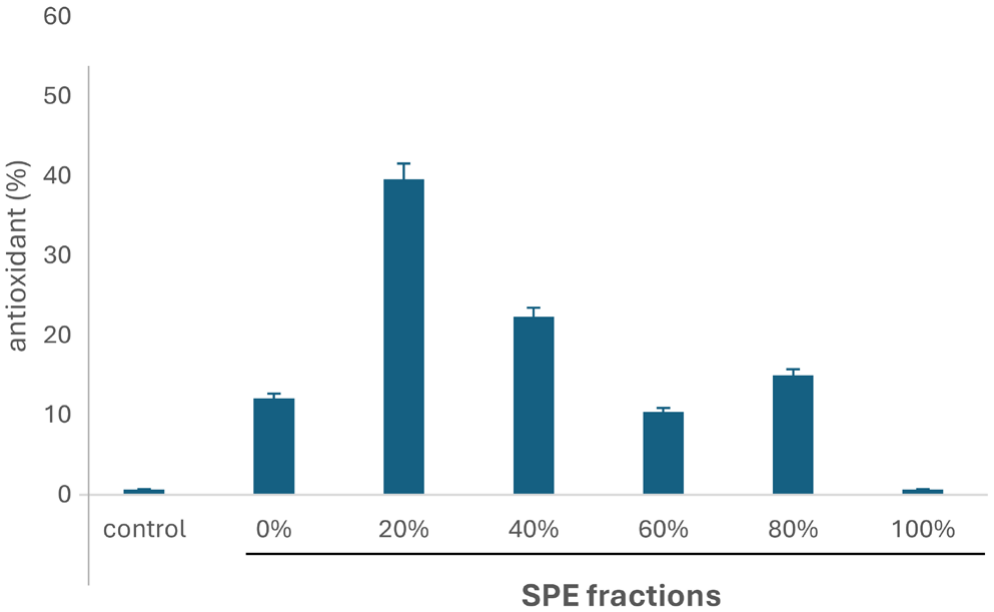
Antioxidant activity (%) of *Tubastraea tagusensis* fractions (1 mg.mL^−1^), obtained by solid phase extraction, assessed by DPPH assay.

The methanolic extract of *Tubastraea tagusensis* was initially tested to verify its antioxidant activity by DPPH, which was confirmed (data not shown). This extract was then submitted to solid‐phase extraction (SPE), in which 6 fractions were generated, according to their acetonitrile percentage (0, 20, 40, 60, 80, and 100%). These fractions were tested by DPPH assay, and fraction 20% had the highest antioxidant activity (Figure 1).

**FIGURE 2:**
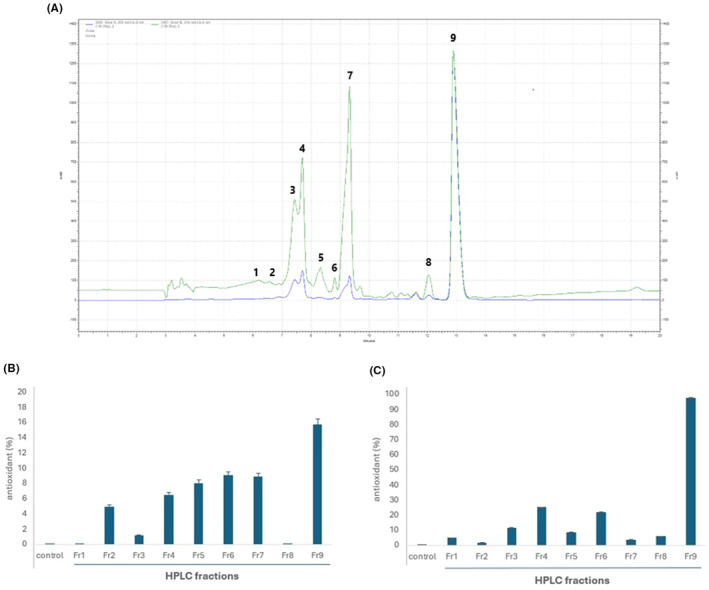
(A) Chromatographic profile of fraction 20%, obtained by SPE, from *Tubastraea tagusensis* methanolic extract. Numbers indicate the peaks collected. The green line is the chromatogram obtained with λ = 214 nm and the blue line 254 nm. (B) DPPH antioxidant assay of 9 fractions from *Tubastraea tagusensis* (1 mg.mL^−*1*^); (C) FRAP antioxidant assay of 9 fractions from *Tubastraea tagusensis* (1 mg.mL^−1^).

The fraction 20% was then selected for further fractionation by HPLC, and the chromatographic profile is shown in Figure 2A. Nine fractions were collected, according to the indications in the figure, and all fractions were tested in antioxidant assays in both DPPH and FRAP methods.

From all fractions, Fr9 presented the highest antioxidant activity in both methods, especially FRAP (Figure 2B,C). This data confirmed its antioxidant capacity in scavenging radicals.

### 3.2 Molecule characterization

**FIGURE 3:**
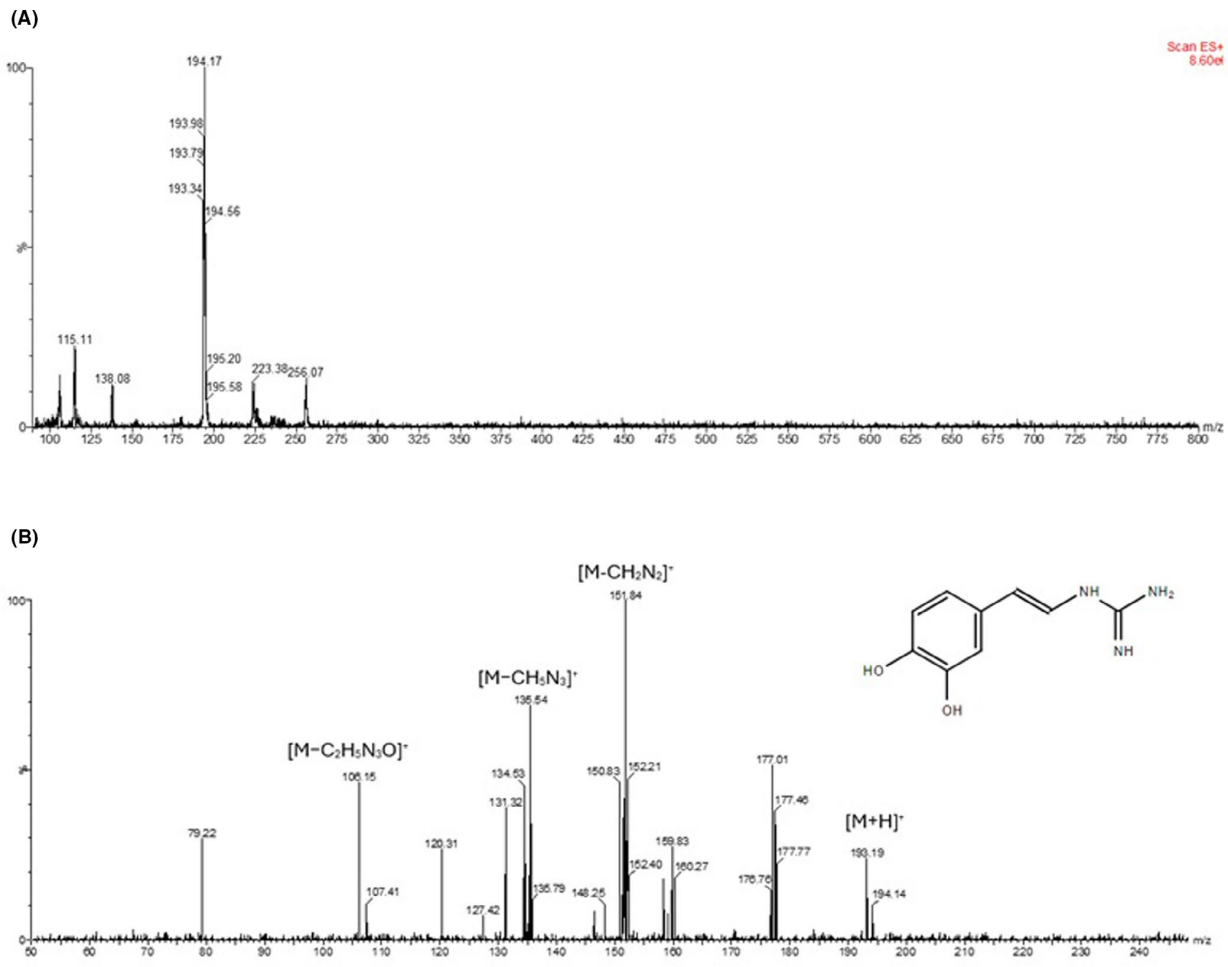
Characterization of tubastrine by mass spectrometry: (A) MS profile of the Fr9 obtained by HPLC; (B) Fragmentation of the ion 194 m/z and MS/MS profile, showing loss of groups, corresponding to tubastrine structure, insert in the figure.

The fraction 9, with antioxidant activity, was analysed by mass spectrometry to initially check its purity. It was observed one main peak in the mass spectrum (Figure 3A), the ion 194.17 m/z. This ion was selected for fragmentation (MS/MS) and the profile of fragment ions is shown in Figure 3B. After a manual analysis and comparison to the fragmentation profile of molecules from *T. tagusensis* described in the literature, we saw that its pattern corresponds to tubastrine, with the loss of groups indicated in the figure, as well as its structure.

### 3.3 Cell viability in AD model

**FIGURE 4:**
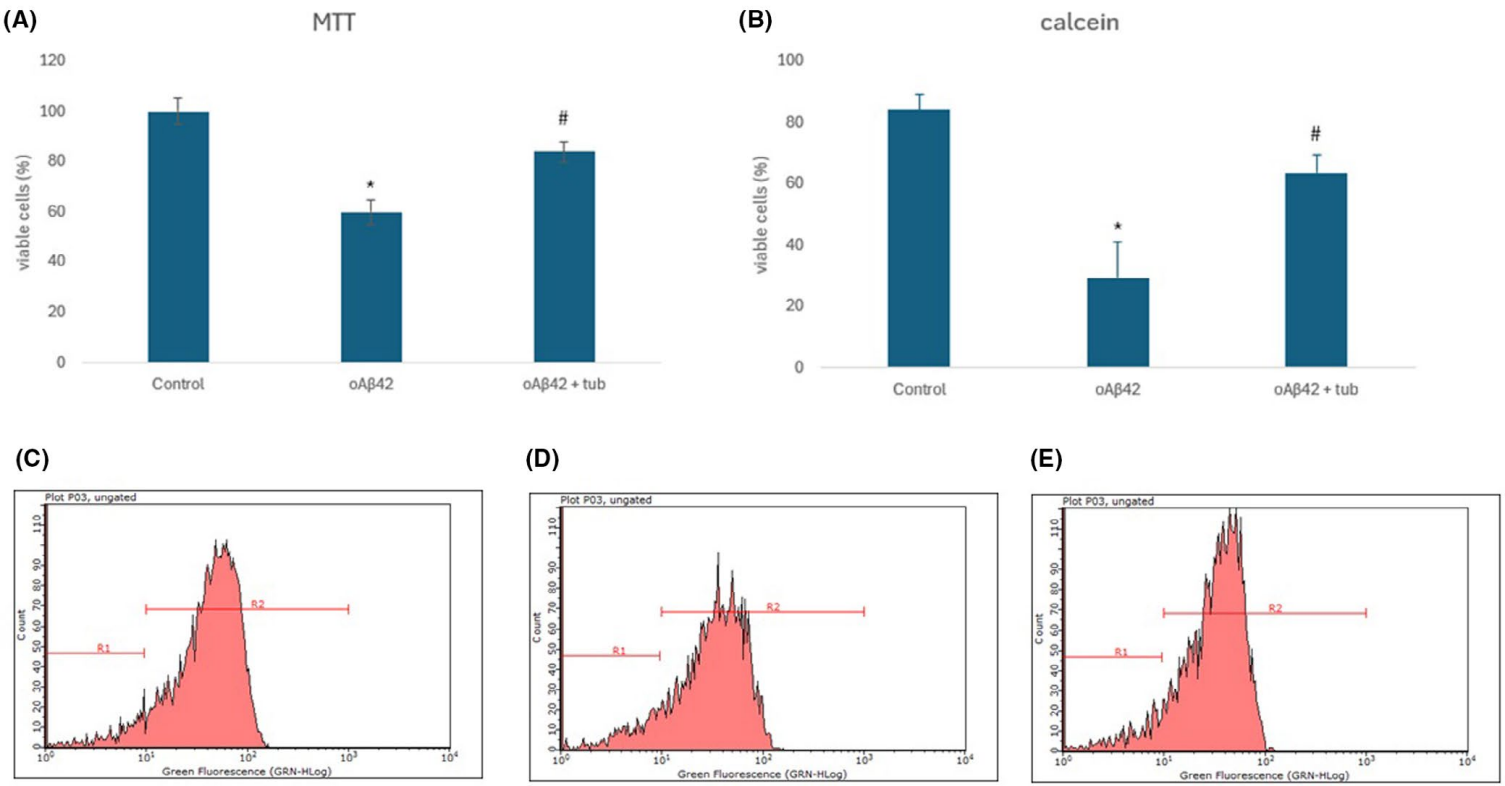
Cell viability evaluation after neuron‐like cells treatment with oAβ42 (5 μM) or oAβ42 (5 μM) followed by tubastrine (100 μM), evaluated by: (A) MTT assay; (B) Viable cells (%) with calcein labeling; (C–E) Flow cytometry histogram of cells analysed with calcein staining and population of cells indicated as R1 or R2 for control, oAβ42 and Aβ42 + tubastrine, respectively. *p < 0.001 between control and oAβ42 and #*p* < 0.001 between oAβ42 and oAβ42 + tubastrine.

To check if the antioxidant activity of tubastrine was enough to inhibit the cell death caused by oAβ42, we performed two assays, MTT and calcein, to evaluate different mechanisms of cell viability. In both assays we could observe that the oAβ42 decreased the number of viable cells, and tubastrine inhibited this cell death (Figure 4).

### 3.4 Antioxidant activity in neurons‐like

**FIGURE 5:**
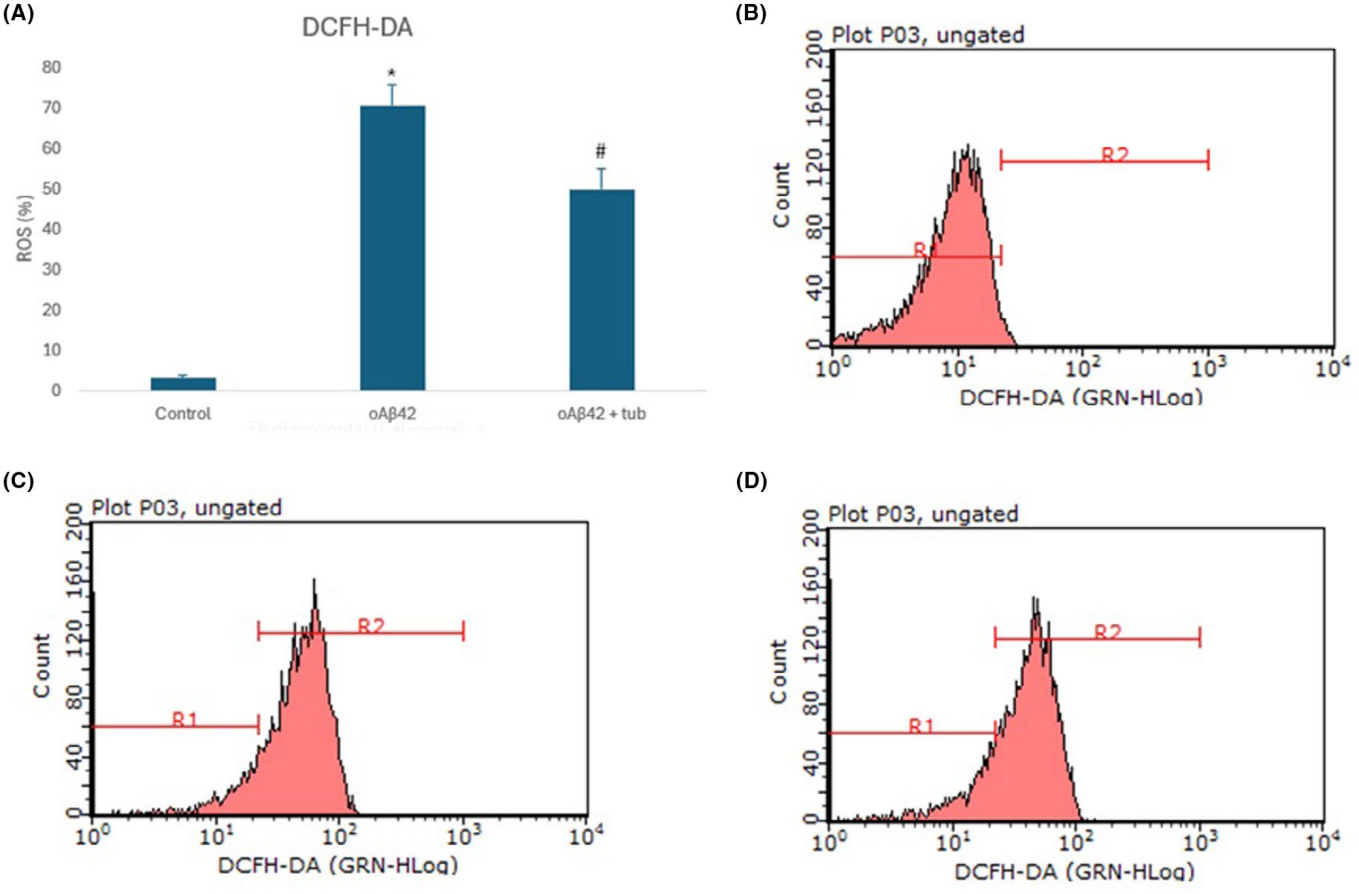
Antioxidant activity of tubastrine (100 μM) on SH‐SY5Y differentiated neurons after exposure to oAβ42 (5 μM): (A) ROS evaluated by DCFH‐DA labeling; (B–D) Flow cytometry histogram of cells analysed and population of cells indicated as R1 or R2 for control, oAβ42 and Aβ42 + tubastrine, respectively. *p < 0.001 between control and oAβ42 and #*p* = 0.007 between oAβ42 and oAβ42 + tubastrine.

**FIGURE 6:**
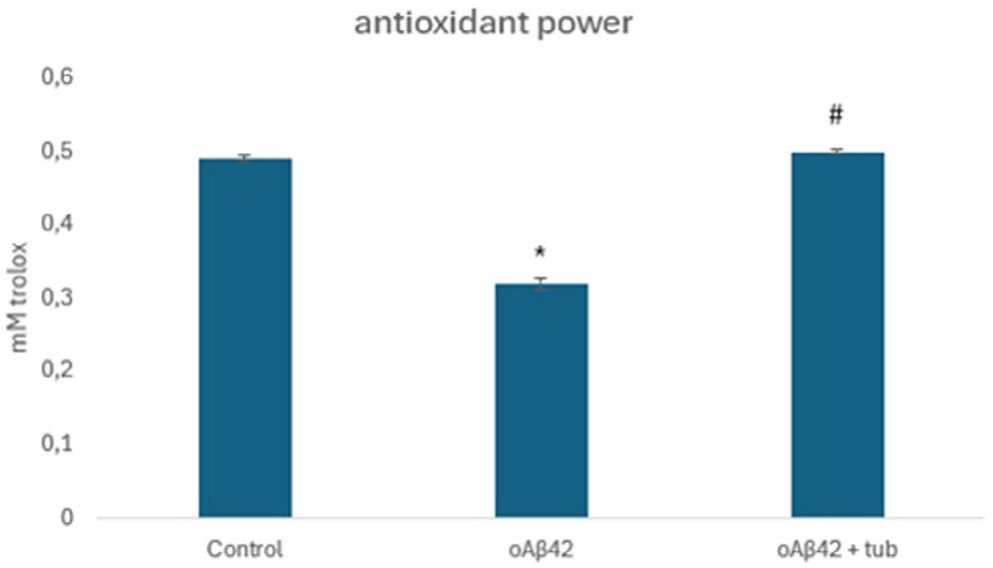
Antioxidant power of neurons‐like by Trolox activity. *p < 0.05 between control and oAβ42 and #*p* = 0.03 between oAβ42 and oAβ42 + tubastrine. #*p* = 0.03 between oAβ42 and oAβ42 + tubastrine.

Neurons‐like SH‐SY5Y cells were incubated with tubastrine, after being exposed to oAβ42. It was possible to detect that the amyloid peptide induced the release of ROS, increasing it to 65% (Figure 5A–C), and the treatment with tubastrine reduced the release of such species to 50% (Figure 5A,D). Similarly, the amyloid peptide reduced the antioxidant power of cells, in comparison to control, and tubastrine recovered the antioxidant capacity, in SH‐SY5Y, as shown in Figure 6.

### 3.5 Molecular docking to evaluate enzymatic antioxidant activity

Tubastrine was evaluated by its capacity to bind in antioxidant enzymes. Superoxide dismutase (SOD), catalase and glutathione peroxidase, the most important ones, were included in such analysis. As shown in Table 1, tubastrine did not bind to SOD and has low binding energy to glutathione peroxidase, indicating an unfavourable interaction. Although a high binding energy was observed with catalase (−7.76 kcal/mol), the position of tubastrine was outside the barrel and far from the loop, including a conserved histidine, important for the catalytic activity. Therefore, in silico approaches indicate low potential of tubastrine in modulating antioxidant enzymes.

**TABLE 1:** Protein targets and binding of tubastrine, evaluated by molecular docking.

Protein	PDB code	Binding energy (kcal/mol)	Binding interaction
Superoxide dismutase	8GSQ (chain A)	−4.250	No binding
Catalase	1DGB (chain A)	−7.765	Arg365, Val74, His75, Asn148
Glutathione peroxidase	2R37 (chain A)	−3.231	Lys137, Asp139

## 4 DISCUSSION

Tubastrine was first isolated by Sakai and Higa (1987)^18^ from *Tubastraea aurea*, extracted with acetone, after being found in the aqueous layer of liquid–liquid extraction with ethyl acetate and then soluble in methanol. In our extraction and fractionation‐based activity, we used similar solvents as this previous study, which reinforces the obtention of tubastrine in our results. Moreover, we isolated a compound from a coral of the same genus as *T. aurea*, so it is not surprising to find tubastrine in both species.

Some *Tubastraea s*sp. are scleractinian corals that invaded the Atlantic and, nowadays, are widespread along the Brazilian coast. Part of its invasiveness success are related to several reproduction traits, as well as their extensive defensive mechanisms, in which antioxidant molecules may be involved.^20^

To confirm the identity of tubastrine, we performed MS and MS/MS analysis, and we could observe the same fragments as the literature: 152, 135, 120, 106, and 103 m/z, besides the exact mass (193.17 Da).^18^, ^21^ By HPLC, we could see that the peak correspondent to tubastrine has high absorbance in 280 nm, similar to that observed by Sakai and Higa, with high absorbance in 287 and 304 nm.

Thus, considering the origin of the molecule, solubility, and solvent extraction, its exact molecular mass, and fragmentation profile, we concluded that our molecule is tubastrine.

Tubastrine (2‐[(E)‐2‐(3,4‐dihydroxyphenyl)ethenyl]guanidine) is an alkaloid guanidinostyrene found in marine animals (Pubchem), other than corals, but few studies explored it from the biochemical point‐of‐view.

Sakai and Higa reported a mild antiviral activity of tubastrine against herpes simplex virus type 1 and vesicular stomatitis virus. Patents of molecules derived from guanidine were deposited, claiming antiviral activity (US4772609A and US‐4851441‐A).

Antibacterial activity against gram‐positive and gram‐negative bacteria was also related to tubastrine, isolated from the marine sponge Spongosorites sp.^22^ Further activity was detected in a bioassay‐guided fractionation, which indicated EGFR inhibition activity from this molecule, isolated from *Dendrodoa grossularia*, an ascidian from the North Sea.^21^

Tubastrine from the ascidian *Aplidium orthium*, as well as some analogues, inhibited the production of superoxide by human neutrophils stimulated with phorbol 12‐myristate 13‐acetate (PMA), besides inhibiting the neutrophil infiltration in vivo. However, they did not cause changes in the xanthine oxidase (XO) enzyme activity, showing no general antioxidant mechanisms.^23^

The enzyme xanthine oxidase catalyses the conversion of hypoxanthine to xanthine, generating superoxide ions and hydrogen peroxide, so considered a source of free radicals.^24^ By molecular docking we evaluated the possible interaction between tubastrine and antioxidant enzymes, and could not see relevant binding, either for low binding energy or molecule positioning. In human SOD, the best binding energy obtained, tubastrine interacted with Arg365, Val74, His75 and Asn148, other than important residues for catalytic activity: His 119 (copper‐binding ligand), His64 (bridging histidine), His72/Gly73 (zinc‐binding ligand), and Arg144 (H_2_O_2_ liganding residue).^25^

Based on these results, we did not evaluate the tubastrine activity on enzymatic mechanism of antioxidation, as the mechanism of its antioxidant activity is radical scavenging.

Antioxidant molecules can act on two pathways: enzymatic and non‐enzymatic. The radical scavenging molecules neutralize free radicals, and can be obtained in minerals, vitamins, organic acids, and cofactors.^26^ They act directly or indirectly after the ROS formation by scavenging its radicals.

We found a radical scavenging activity of the molecule in both DPPH and FRAP assays. DPPH assay consists of the monitoring of a change of the radical reagent to a reduced form in the presence of a molecule capable of donating hydrogen atoms,^27^ and FRAP measures the reduction of ferric ion (Fe^3+^) to Fe^2+^ complexed by an antioxidant molecule in an acidic medium. Thus, tubastrine can form a complex with metal atoms, important to maintain electrons in the mitochondrial electron transport chain.^28^

Tubastrine has two hydroxyl groups, which donate these atoms and consequently scavenge the free radicals (ROS). This capacity was observed in two approaches (DPPH and FRAP), in which the reagents were reduced in the presence of tubastrine.

Using the same method, we could identify an antioxidant activity among 30 glucal‐based triazoles analogues.^29^ The molecule with the best activity was a triazole containing three‐OH functional groups, which is known to favour radical scavenging, similar to tubastrine. Thus, we confirmed that the mechanism of antioxidant activity of tubastrine in due to non‐enzymatic pathway.

The incubation of oAβ42 induced the release of H_2_O_2_ from SH‐SY5Y cell line, in agreement with the literature,^30^ which shows the success of our cellular model. SH‐SY5Y has been widely used in studies that focus on neurodegenerative diseases, as its differentiation with retinoic acid changes the cells' morphology such as increase of neuritic process, electrical excitability, and synaptophysin‐positive synapses, characteristics of cholinergic and dopaminergic neurons.^31^, ^32^

The radical scavenging capacity of tubastrine was sufficient to decrease ROS release from the cells, induced by the amyloid peptide. Besides, the molecule increased the total antioxidant capacity of cells, by measuring its ability to inhibit the oxidation of ABTS. These data confirm its efficacy of radical scavenging in a cell culture, which shows a possibility for further studies that focus on its application for AD.

Moreover, such antioxidant activity of tubastrine on neurons‐like was able to reduce the cells's death induced by oAβ42, considering that ROS released from the mitochondria contributes to cell apoptosis. This activity was measured by two assays: calcein and MTT. Calcein evaluates the cell membrane integrity, and the viable cells based on the cell disruption. On the other hand, in the MTT assay, the number of viable cells is estimated based on the mitochondrial activity of living cells, on the cleavage of the tetrazolium ring by dehydrogenases. So, in that case, besides cell viability, we could also evaluate the mitochondrial activity, which was recovered after tubastrine treatment, reinforcing the importance of the molecule in reducing ROS formation. Nevertheless, cell death mechanisms should be further explored.

We have seen similar results with a fraction from the *Echinometra lucunter* sea urchin. A 50% SPE extract contains antioxidant compounds that could scavenge radicals and reduce peroxide formation. This effect, added to other mechanisms, caused the prevention and inhibition of cell death.^32^

Thus, these data show that marine animals can provide interesting compounds that interfere with toxic effects caused by oAβ42, representing an important source for bioactive molecules that can be useful for the development of new therapeutical prototypes, aiming the treatment of AD.

In conclusion, in an antioxidant activity‐guided fractionation, we isolated tubastrine from the scleractinian coral *T. tagusensis*, which was able to scavenge free radicals and reduce the oxidative stress in neurons‐like, an important pathophysiological mechanism of AD contributing to the reversion of toxic effects caused by the amyloid peptide.

## AUTHOR CONTRIBUTIONS

AGS: Methodology, Investigation; JVCR: Investigation; FDR: Investigation; FDS: Investigation; JGP: Investigation; MVK: Formal Analysis, Resources; GBL: Methodology, Formal Analysis; JMS: Conceptualization, Formal Analysis, Funding Acquisition, Supervision, Writing: Original Draft Preparation; all authors: Writing – Review & Editing.

## FUNDING INFORMATION

This research was funded by FAPESP, grant number 2019/19929‐6.

## CONFLICT OF INTEREST STATEMENT

The authors declare no conflicts of interest.

## Data Availability

Data available on request from the authors.

## References

[1] Betteridge DJ . What is oxidative stress? Metabolism. 2000;49(2):3‐8. 10.1016/s0026-0495(00)80077-3 10693912

[2] Cui H , Kong Y , Zhang H . Oxidative stress, mitochondrial dysfunction, and aging. J Signal Transduct. 2012;2012:1‐13. 10.1155/2012/646354 PMC3184498 21977319

[3] Khan S , Barve KH , Kumar MS . Recent advancements in pathogenesis, diagnostics and treatment of Alzheimer's disease. Curr Neuropharmacol. 2020;18(11):1106‐1125. 32484110 10.2174/1570159X18666200528142429 PMC7709159

[4] Crous‐Bou M , Minguillón C , Gramunt N , Molinuevo JL . Alzheimer's disease prevention: from risk factors to early intervention. Alzheimers Res Ther. 2017;9(1):71. 28899416 10.1186/s13195-017-0297-z PMC5596480

[5] Scheltens P , De Strooper B , Kivipelto M , et al. Alzheimer's disease. Lancet. 2021;397(10284):1577‐1590. 33667416 10.1016/S0140-6736(20)32205-4 PMC8354300

[6] Grimm MO , Hartmann T . Recent understanding of the molecular mechanisms of Alzheimer?s disease. J Addict Res Ther. 2011;s5:004.

[7] Reddy PH , Oliver DM . Amyloid Beta and Phosphorylated tau‐induced defective autophagy and mitophagy in Alzheimer's disease. Cells. 2019;8(5):488. 31121890 10.3390/cells8050488 PMC6562604

[8] Perez FP , Bose D , Maloney B , Nho K , Shah K , Lahiri DK . Late‐onset Alzheimer's disease, heating up and foxed by several proteins: pathomolecular effects of the aging process. J Alzheimers Dis. 2014;40(1):1‐17. 24326519 10.3233/JAD-131544 PMC4126605

[9] Ionescu‐Tucker A , Cotman CW . Emerging roles of oxidative stress in brain aging and Alzheimer's disease. Neurobiol Aging. 2021;107:86‐95. 34416493 10.1016/j.neurobiolaging.2021.07.014

[10] Leuner K , Schütt T , Kurz C , et al. Mitochondrion‐derived reactive oxygen species Lead to enhanced amyloid Beta formation. Antioxid Redox Signal. 2012;16(12):1421‐1433. 22229260 10.1089/ars.2011.4173 PMC3329950

[11] Wojsiat J , Zoltowska KM , Laskowska‐Kaszub K , Wojda U . Oxidant/antioxidant imbalance in Alzheimer's disease: therapeutic and diagnostic prospects. Oxidative Med Cell Longev. 2018;2018:1‐16. 10.1155/2018/6435861 PMC5831771 29636850

[12] Mazzanti G , Di Giacomo S . Curcumin and resveratrol in the Management of Cognitive Disorders: what is the clinical evidence? Molecules. 2016;21(9):1243. 27649135 10.3390/molecules21091243 PMC6273006

[13] Grimm M , Mett J , Hartmann T . The impact of vitamin E and other fat‐soluble vitamins on Alzheimer's disease. Int J Mol Sci. 2016;17(11):1785. 27792188 10.3390/ijms17111785 PMC5133786

[14] Coelho GR , da Silva DL , Beraldo‐Neto E , et al. Neglected venomous animals and toxins: underrated biotechnological tools in drug development. Toxins (Basel). 2021;13(12):851. 34941689 10.3390/toxins13120851 PMC8708286

[15] Miljanich GP . Ziconotide: neuronal Calcium Channel blocker for treating severe chronic pain. Curr Med Chem. 2004;11(23):3029‐3040. 15578997 10.2174/0929867043363884

[16] Schwartsmann G , da Rocha AB , Berlinck RG , Jimeno J . Marine organisms as a source of new anticancer agents. Lancet Oncol. 2001;2(4):221‐225. 11905767 10.1016/s1470-2045(00)00292-8

[17] Carter NJ , Keam SJ . Trabectedin. Drugs. 2007;67(15):2257‐2276. 17927287 10.2165/00003495-200767150-00009

[18] Sakai R , Higa T . Tubastrine, a new guanidinostyrene from the coral *Tubastrea aurea* . Chem Lett. 1987;16(1):127‐128.

[19] Banagouro KCQ , Viana J , de Lima LP , et al. Biochemical and Toxinological characterization of venom from Macrorhynchia philippina (cnidaria, hydrozoa). Biomed Res Int. 2022;2022:1‐10. 10.1155/2022/8170252 PMC9129954 35620224

[20] Luz BLP , Di Domenico M , Migotto AE , Kitahara MV . Life‐history traits of *Tubastraea coccinea* : reproduction, development, and larval competence. Ecol Evol. 2020;10(13):6223‐6238. 32724509 10.1002/ece3.6346 PMC7381571

[21] Barenbrock JS , Köck M . Screening enzyme‐inhibitory activity in several ascidian species from Orkney Islands using protein tyrosine kinase (PTK) bioassay‐guided fractionation. J Biotechnol. 2005;117(3):225‐232. 15862352 10.1016/j.jbiotec.2005.01.009

[22] Urban S , Capon R , Hooper J . A new alkaloid from an Australian marine sponge, Spongosorites sp. Aust J Chem. 1994;47(12):2279.

[23] Pearce AN , Chia EW , Berridge MV , et al. Orthidines A–E, tubastrine, 3,4‐dimethoxyphenethyl‐β‐guanidine, and 1,14‐sperminedihomovanillamide: potential anti‐inflammatory alkaloids isolated from the New Zealand ascidian Aplidium orthium that act as inhibitors of neutrophil respiratory burst. Tetrahedron. 2008;64(24):5748‐5755.

[24] Bortolotti M , Polito L , Battelli MG , Bolognesi A . Xanthine oxidoreductase: one enzyme for multiple physiological tasks. Redox Biol. 2021;41:101882. 33578127 10.1016/j.redox.2021.101882 PMC7879036

[25] Perry JJP , Shin DS , Getzoff ED , Tainer JA . The structural biochemistry of the superoxide dismutases. Biochimica et Biophysica Acta (BBA)—Proteins and Proteomics. 2010;1804(2):245‐262. 19914407 10.1016/j.bbapap.2009.11.004 PMC3098211

[26] Carocho M , Ferreira ICFR . A review on antioxidants, prooxidants and related controversy: natural and synthetic compounds, screening and analysis methodologies and future perspectives. Food Chem Toxicol. 2013;51:15‐25. 23017782 10.1016/j.fct.2012.09.021

[27] Gulcin İ , Alwasel SH . DPPH radical scavenging assay. PRO. 2023;11(8):2248.

[28] Benzie IFF , Strain JJ . The ferric reducing ability of plasma (FRAP) as a measure of “antioxidant power”: the FRAP assay. Anal Biochem. 1996;239(1):70‐76. 8660627 10.1006/abio.1996.0292

[29] Vieira Veloso R , Shamim A , Lamarrey Y , Stefani HA , Mozer Sciani J . Antioxidant and anti‐sickling activity of glucal‐based triazoles compounds—an in vitro and in silico study. Bioorg Chem. 2021;109:104709. 33636439 10.1016/j.bioorg.2021.104709

[30] Casley CS , Canevari L , Land JM , Clark JB , Sharpe MA . β‐Amyloid inhibits integrated mitochondrial respiration and key enzyme activities. J Neurochem. 2002;80(1):91‐100. 11796747 10.1046/j.0022-3042.2001.00681.x

[31] Kovalevich J , Langford D . Considerations for the use of SH‐SY5Y neuroblastoma cells in. Neurobiology. 2013;9‐21:1078. 10.1007/978-1-62703-640-5_2 PMC5127451 23975817

[32] da Silva AG , Alves MDM , da Cunha AA , et al. Echinometra lucunter molecules reduce Aβ42‐induced neurotoxicity in SH‐SY5Y neuron‐like cells: effects on disaggregation and oxidative stress. J Venom Anim Toxins Incl Trop Dis. 2023;29:e20230031. 38053575 10.1590/1678-9199-JVATITD-2023-0031 PMC10694836

